# Clinical Knowledge Supported Acute Kidney Injury (AKI) Risk Assessment Model for Elderly Patients

**DOI:** 10.3390/ijerph18041607

**Published:** 2021-02-08

**Authors:** Kao-Yi Shen, Yen-Ching Chuang, Tao-Hsin Tung

**Affiliations:** 1 Department of Banking & Finance, Chinese Culture University, Taipei 11114, Taiwan; 2 Taiwan Association of Health Industry Management and Development, Taipei 10351, Taiwan; yenching.chuang@gmail.com; 3 Evidence-Based Medicine Center, Taizhou Hospital of Zhejiang Province Affiliated to Wenzhou Medical University, Linhai 317000, China; ch2876@yeah.net

**Keywords:** acute kidney injury (AKI), geriatric, frailty, meta-analysis, multiple criteria decision-making (MCDM), group decision

## Abstract

From the clinical viewpoint, the statistical approach is still the cornerstone for exploring many diseases. This study was conducted to explore the risk factors related to acute kidney injury (AKI) for elderly patients using the multiple criteria decision-making (MCDM) approach. Ten nephrologists from a teaching hospital in Taipei took part in forming the AKI risk assessment model. The key findings are: (1) Comorbidity and Laboratory Values would influence Comprehensive Geriatric Assessment; (2) Frailty is the highest influential AKI risk factor for elderly patients; and (3) Elderly patients could enhance their daily activities and nutrition to improve frailty and lower AKI risk. Furthermore, we illustrate how to apply MCDM methods to retrieve clinical experience from seasoned doctors, which may serve as a knowledge-based system to support clinical prognoses. In conclusion, this study has shed light on integrating multiple research approaches to assist medical decision-making in clinical practice.

## 1. Introduction

There are roughly two types of clinical research methods in conventional medical research: observation-based statistical testing and meta-analysis [1]. The former has to collect over one group of data, after controlling for demographic factors and the associated variables (e.g., symptoms) of patients, to examine the premises or consequences of a targeted illness (or disease). The latter stands on the grounds of published homogeneous research to explore hypothesized patterns from the papers that satisfy the inclusion criteria [1,2]. They both belong to the evidence-based statistical approach, and we focus on enhancing the findings of a meta-analysis in this work.

Though the statistical approach has been broadly adopted, certain limitations are rooted in the presumptions of statistics [3]. For instance, most statistical models assume variables to be independent, which is controversial. Besides, researchers have to presume the probabilistic distribution(s) of involved variables. It is difficult to validate those assumptions [4]; those limitations impede researchers to explore complicated relations in their models. Furthermore, there is nearly no room to accommodate the clinical experiences/knowledge from doctors. The research gap between the findings of meta-analysis and clinical prognoses exists.

In clinical research, the statistical approach is still the cornerstone to understanding many illnesses. A well-devised meta-analysis may explore and examine inconclusive disease patterns—with rigorous control and testing—from credible sources. Its contributions are evident. However, owing to the limitations of its modeling methods (i.e., statistics), meta-analysis is insufficient for clarifying the influential relationship among a group of factors without presuming the probabilistic distributions of its variables. In most cases, meta-analysis can merely identify the significance of one or more factors that might be associated with a disease. Therefore, to enrich a meta-analysis’ findings and bridge the gap, this study proposes the multiple criteria decision-making (MCDM) approach [5,6,7] to incorporate clinical knowledge from seasoned doctors.

Unlike traditional statistics, the MCDM approach can support decision-makers (DMs) or experts to make rational judgments by considering multiple factors simultaneously, without presumed probabilistic distributions. This approach hinges upon domain experts’ knowledge/experience. Certain MCDM methods may unveil the convoluted interrelationship of a complex model, such as Decision-Making Trial and Evaluation Laboratory (DEMATEL) [8,9], Analytical Network Process (ANP) [10], and the DEMATEL-based ANP (DANP) [11,12]. The advantages of MCDM methods complement the limitations of meta-analyses; the outcome may even serve as a knowledge-based system to support clinical prognoses.

The present study adopts acute kidney injury (AKI) [13] as an empirical case, which has a high risk of mortality for the elderly. By definition, “AKI is an abrupt decrease in kidney function that includes, but is not limited to, acute renal failure. [14]” Nowadays, AKI is becoming common among elderly patients, who are more vulnerable to kidney failure or dysfunction [15]. In this context, our research purposes are twofold: (1) elaborate the findings from a published meta-analysis based on the clinical experience of doctors and (2) construct a flexible knowledge-based system to support clinical prognoses. The specific aims of this case study are to:

(1) explore the interrelationships between the risk factors that might induce AKI in elderly patients;
(2) identify the relative influence of each criterion on the risk assessment of AKI;
(3) provide precautionary guidance for elderly patients to reduce their potential AKI. risk.

Aside from the plausible advantages, the proposed model has the flexibility to adjust the weighting of doctors’ opinions based on their experience (or expertise in a specific field). This flexibility is yielded from the group decision theory [16]. This advantage contributes to carrying out the policy of a hospital. Evaluating a patient with complicated symptoms or comorbidity usually requires doctors’ participation with different experience levels and varied expertise. The hybrid MCDM approach can fuse experts’ knowledge with a customized weighting policy. This is another contribution that the present study might bring to clinical applications. We will illustrate this idea by an example in Section 4.

This paper is divided into five sections. Section 1 discusses the background information as above. Section 2 provides the research material and the proposed hybrid MCDM model. Section 3 reports the modeling of AKI risk assessments for elderly patients. Section 4 provides discussions with an illustration of how to apply the model and Section 5 concludes this study.

## 2. Materials and Research Methods

This section explains the materials adopted in modeling. Since the present study aims to enrich meta-analysis findings, a study of AKI in the elderly population from a recently published paper [15] serves as the modeling foundation. Therefore, in Section 3.1, the findings of [15] are briefly highlighted. Besides this, ten experienced doctors took part in this work. Section 2.2 introduces two MCDM methods: the DEMATEL technique [8,9] and the DEMATEL-based ANP (DANP) [11,12]. The first one can indicate the influential relationship of a model with multiple factors (criteria). The second one may explore the relative influence of each factor on supporting clinical prognoses.

### 2.1. Materials

The meta-analysis article [15] that we referred to mainly discussed whether elderly patients might suffer from a higher risk of AKI because of frailty. It began with 1385 papers that are included in PubMed (566), the Cochrane Library (609), and EMBASE (210). After following the PRISMA (Preferred Reporting Items for Systematic Review and Meta Analysis) research flow [17], it excluded duplicated, unrelated or unsuitable articles. It eventually identified four cohort studies [18,19,20,21] that met the inclusion criteria. Its rigorous research process [15] attempted to overcome the potential issues of meta-analysis (such as low quality of included studies and heterogeneity [22]), which serves as the cornerstone of the present study.

Owing to its research target, the meta-analysis [15] attempted to include studies that controlled for the variable, diagnosed AKI or non-AKI, for those elderly with frailty. The selection process examined the inter-rater reliability and evaluated the degree of variation across studies; both passed the associated statistical thresholds (i.e., the kappa and $I^{2}$ statistics). Among the four cohort studies, the study duration ranged from two weeks [20] to four years [18]. The total subjects included in each study were between 164 [20] to 533 [19].

Though the four studies adopted different frailty measures (e.g., Clinical Frailty Scores), their assessments on frailty are all grounded on clinically recognized measurements. The meta-analysis from the four papers confirmed the significant association between frailty and AKI among elderly patients.

### 2.2. Research Methods

**Figure 1:**
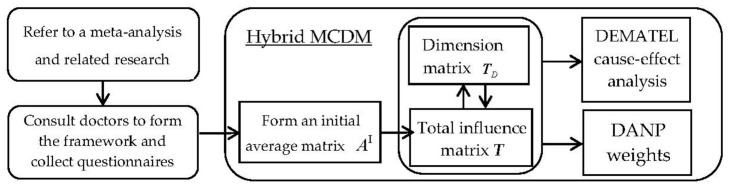
Research flow of the hybrid MCDM (multiple criteria decision-making) model.

In this subsection, we introduce the essentials of the DEMATEL and DEMATEL-based ANP (DANP). DANP is extended from the DEMATEL, which adopts the central idea of ANP to derive the relative influence of each criterion of a hybrid decision model. The required research flows are illustrated in Figure 1.

#### 2.2.1. DEMATEL Technique

The DEMATEL technique was devised to resolve complicated issues, and assumes that all the included factors (criteria) are interrelated. Furthermore, it relies on domain experts’ knowledge (opinions) to derive the influential relationship among a model’s factors. The essential steps to conduct the DEMATEL analysis are as follows.

**Step 1**: Collect opinions from domain experts to form an initial matrix $A^{I}$.

As mentioned above, the DEMATEL technique presumes that all the included criteria are interrelated. Therefore, it begins by devising a questionnaire to have all the criteria with questions like: “in your opinion, what is the influence of criterion *i* on criterion *j* (the answer ranges from “0 (No Influence),” “1 (Minor Influence),” “2 (Moderate Influence)” to “3 (High Influence),” to “4 (Very High Influence)).” After collecting opinions from domain experts, the averaged result of the influence of criterion *i* on criterion *j* will be indicated as $\bar{a_{ij}}$ in $A^{I}$ ($A^{I}={\left[\bar{a_{ij}}\right]}_{n\times n}$, for *i*,*j* = 1,…,*n*), located on the *i*-th row and the *j*-th column of $A^{I}$.

**Step 2**: Normalize $A^{I}$ to become a matrix N.

The normalized matrix N can be obtained by calculating *k*, where $k=\max\left\{\max\sum_{j=1}^{n}\bar{a_{ij}},\max\sum_{i=1}^{n}\bar{a_{ij}}\right\}$, and the normalized matrix $N=k\times A^{I}$.

**Step 3**: Calculate the total-influence matrix T.

The total-influence matrix T is defined as $T=N+N^{2}+\ldots +N^{w}$, which indicates the chain influence by raising the number of *w*. It can be decomposed as Equation (1):

(1) $$T=N\times (I-N^{w})\times {\left(I-N\right)}^{-1}$$

while w→∞, $N^{w}≅{\left[0\right]}_{n\times n}$. Therefore, the total-influence matrix T can be approximated and indicated as: $T=N\times {\left(I-N\right)}^{-1}={\left[t_{ij}\right]}_{{}_{n\times n}}$. The sum of each row and column of T can form two vectors, termed as $r^{C}={\left(r_{1},\cdots ,r_{i},\cdots ,r_{n}\right)}^{\prime }$ and $d^{C}=\left(d_{1},\cdots ,d_{j},\cdots ,d_{n}\right)$. In the DEMATEL analytic, “$r^{C}+d^{C}$” and “$r^{C}-d^{C}$” can be adopted to indicate the directional influence relationship among criteria. The details will be explained in Section 4.

**Step 4**: Form initial unweighted super-matrix $T_{C}^{S}$.

Assume that there are *m* dimensions in T. Then, the total-influence matrix T can be partitioned by its dimensions and indicated as an initial unweighted super-matrix $T_{C}^{S}$ that comprises m×m sub-matrices in Equation (2).

(2) $$T_{C}^{S}=\begin{array}{cc} & \begin{array}{ccccc} D_{1}\;\;\; & & D_{j}\;\;\; & & D_{m}\;\;\; \end{array}\\ \begin{array}{c} D_{1}\;\;\\ \;\;\vdots \;\;\\ \;\;D_{i}\;\;\\ \;\;\vdots \;\;\\ \;\;D_{m} \end{array} & {\left[\begin{array}{ccccc} T_{C}^{S11} & \cdots & T_{C}^{S1j} & \cdots & T_{C}^{S1m}\\ \vdots & & \vdots & & \vdots \\ T_{C}^{Si1} & \cdots & T_{C}^{Sij} & \cdots & T_{C}^{Sim}\\ \vdots & & \vdots & & \vdots \\ T_{C}^{Sm1} & \cdots & T_{C}^{Smj} & \cdots & T_{C}^{Smm} \end{array}\right]}_{n\times n} \end{array},\mathrm{for}1<i,j\leq m<n.$$

For instance, if there were three criteria in $D_{1}$, the sub-matrix $T_{C}^{S11}$ would be a 3×3 matrix. $T_{C}^{S11}$ denotes the corresponding upper-left 3×3 elements of $T_{C}^{S}$.

**Step 5**: Form the dimension matrix $T_{D}^{}$.

The averaged figures of all elements of each sub-matrix of $T_{C}^{S}$ will form a m×m dimension matrix, which is shown in Equation (3):

(3) $$T_{D}={\left[\begin{array}{ccccc} \bar{t_{C}^{S11}} & \cdots & \bar{t_{C}^{S1j}} & \cdots & \bar{t_{C}^{S1m}}\\ \vdots & & \vdots & & \vdots \\ \bar{t_{C}^{Si1}} & \cdots & \bar{t_{C}^{Sij}} & \cdots & \bar{t_{C}^{Sim}}\\ \vdots & & \vdots & & \vdots \\ \bar{t_{C}^{Sm1}} & \cdots & \bar{t_{C}^{Smj}} & \cdots & \bar{t_{C}^{Smm}} \end{array}\right]}_{m\times m},\mathrm{for}1<i,j\leq m.$$

Similarly, the sum of each row and column of $T_{D}^{N}$ can form two vectors, termed as $r^{D}={\left(r_{1}^{D},\cdots ,r_{i}{}^{D},\cdots ,r_{m}^{D}\right)}^{\prime }$ and $d^{D}=\left(d_{1}{}^{D},\cdots ,d_{j}^{D},\cdots ,d_{m}^{D}\right)$. Then, “$r^{D}+d^{D}$” and “$r^{D}-d^{D}$” can be adopted to indicate the directional influence relationship among the dimensions. Based on the DEMATEL analytics from Steps 3 and 5, an Internetwork Relationship Map (INRM) can help depict the directional influences among dimensions and criteria.

#### 2.2.2. DEMATEL-Based ANP (DANP)

The DANP method adopts the central idea of the ANP and the transpose of $T_{C}^{S}$ (i.e., $W={\left(T_{C}^{S}\right)}^{\prime }$) is regarded as an unweighted supermatrix in the ANP. Compared with the ANP’s assumption, the DANP considers the dimensional influences while forming an initial supermatrix.

**Step 6**: Obtain the initial supermatix of DANP.

First, the dimension matrix $T_{D}^{}$ from **Step 5** should be normalized to become $T_{D}^{N}$ (refer to Equation (4)). Then, the initial supermatrix of DANP can be obtained by using $T_{D}^{N}$ to adjust the unweighted supermatrix W(i.e., $W^{adj}=T_{D}^{N}\times W$).

(4) $$T_{D}^{N}={\left[\begin{array}{ccccc} \bar{t_{C}^{S11}}/\sum_{j=1}^{m}\bar{t_{C}^{S1j}} & \cdots & \bar{t_{C}^{S1j}}/\sum_{j=1}^{m}\bar{t_{C}^{S1j}} & \cdots & \bar{t_{C}^{S1m}}/\sum_{j=1}^{m}\bar{t_{C}^{S1j}}\\ \vdots & & \vdots & & \vdots \\ \bar{t_{C}^{Si1}}/\sum_{j=1}^{m}\bar{t_{C}^{Sij}} & \cdots & \bar{t_{C}^{Sij}}/\sum_{j=1}^{m}\bar{t_{C}^{Sij}} & \cdots & \bar{t_{C}^{Sim}}/\sum_{j=1}^{m}\bar{t_{C}^{Sij}}\\ \vdots & & \vdots & & \vdots \\ \bar{t_{C}^{Sm1}}/\sum_{j=1}^{m}\bar{t_{C}^{Smj}} & \cdots & \bar{t_{C}^{Smj}}/\sum_{j=1}^{m}\bar{t_{C}^{Smj}} & \cdots & \bar{t_{C}^{Smm}}/\sum_{j=1}^{m}\bar{t_{C}^{Smj}} \end{array}\right]}_{m\times m},\mathrm{for}1<i,j\leq m.$$

Once the adjusted supermatrix $W^{adj}$ is obtained, the influential weights can be reached by raising the number of *q* in $\underset{q\to \infty }{\lim}{\left(W^{Adj}\right)}^{q}$ until $W^{adj}$ becomes stable. After normalizing the stable weights, the final DANP influential weights can be obtained.

## 3. AKI Risk Assessment Model for Elderly Patients

In the present study, the definition of AKI is based on Kidney Disease: Improving Global Outcomes (KDIGO), and the criterion for elderly patients is aged ≥65 years. Due to AKI being a complex disorder related to the interplay of patient-associated factors within the environment where patients live, the dimensions of heterogeneous factors should consider environmental exposures and the inherent risk elements [23]. In each dimension, risk factors could be identified to design and implement preventive interventions or further investigations.

In this context, the preliminary version of the AKI risk assessment model is based on the meta-analysis article [15] and related research (i.e., [18,19,20,21], summarized in Table 1), and the three dimensions all come from [19] after removing demographic factors. One thing that needs to be mentioned here is that Frailty, (***C***_33_), is the primary factor highlighted by [15].

Next, we further consulted on the preliminary version with ten nephrologists from one teaching hospital in Taipei. Based on their clinical experience, they suggested including another two criteria: “Depression (***C*_13_**)” and “Hepatitis B/C (***C*_15_**)” for AKI elderly patients. After several rounds of discussions with the doctors, we finalized the 14 criteria in three dimensions, shown in Table 2.

This study’s purposive sampling was conducted in one medical center in northern Taiwan from April 2020 to June 2020. We recruited well-trained nephrologists who were able to express willingness to participate in the survey.

In Table 2, the three dimensions all come from [19] after removing demographic factors. The estimated glomerular filtration rate (eGFR) is based on the CKD epidemiology collaboration formula [24]. 

### 3.1. DEMATEL Analysis

From Steps 1 to 4 in the previous section, we collected the ten doctors’ opinions. In this case, to mitigate the potential risk of outliers that might distort the average result, this study adopted the median value, instead of the arithmetic mean, to form the initial average matrix $A^{I}$ (Table 3).

**Figure 2:**
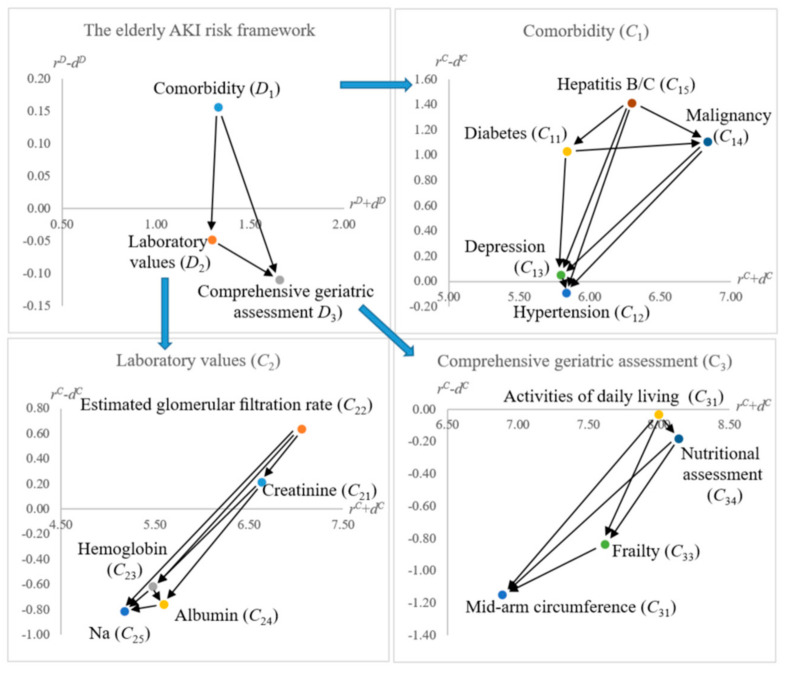
Internetwork relationship map (INRM).

Since there are three dimensions (i.e., m=3) in this analysis, the dimension matrix $T_{D}^{}$ is a 3×3 matrix, shown in Appendix A (Table A1). The associated directional influence analytics for the dimensions and criteria are summarized in Table 4 and illustrated in Figure 2.

The opinions that comprise $A^{I}$ (i.e., Table 3) were examined by Equation (5). The confidence level (${\mathrm{CL}}_{A^{I}}$) for the result is higher than 95% and *k* is the number of questionnaires (*k* = 10 in here). In other words, the initial average matrix carries enough confidence to indicate the averaged opinions to forming $A^{I}$.

(5) $${\mathrm{CL}}_{A^{I}}=\frac{1}{n(n-1)}\sum_{i=1}^{k}\sum_{j=1}^{k}\frac{\left|\bar{a_{ij}^{k}}-\bar{a_{ij}^{k-1}}\right|}{\bar{a_{ij}^{k}}}\times 100\%$$

From Table 4, ***D*_1_** (Comorbidity) would influence ***D*_2_** (Laboratory Values) and ***D*_3_** (Comprehensive Geriatric Assessments). It is worthwhile to delve into ***D*_3_**, which indicates that Mid-arm circumference (***C*_32_**) and Frailty (***C*_33_**) are influenced by Activities of daily living (***C*_31_**) and Nutritional assessment (***C*_34_**).

### 3.2. DEMATEL-Based ANP (DANP) Weights

Based on the initial average matrix $A^{I}$ in Table 3 and the six steps in Section 2, we obtained the DANP influential weights for the AKI risk assessment model. The normalized dimensional matrix $T_{D}^{N}$ is shown in Table 5, and the associated influential weight of each criterion is summarized in Table 5. The DEMATEL-adjusted initial supermatrix is in Appendix A (Table A2).

In Table 6, ***D*_3_** (Comprehensive Geriatric Assessments) shows the highest influential weight (i.e., 41.17%), and Frailty (***C*_33_**) is the most influential criterion. This finding echoes the conclusion of [15]. Though the result is similar to the previous research [15,18,20], the DEMATEL analysis further identifies the source factors (dimensions/criteria) of AKI for the elderly patients. In Figure 1, Comorbidity (***D*_1_**) and Laboratory Values (***D*_2_**) both would influence ***D*_3_** (Comprehensive Geriatric Assessments). Besides, within ***D*_3_** (Comprehensive Geriatric Assessments), the DEMATEL analysis suggests that ***C*_31_** (Activities of daily living) and ***C*_34_**(Nutritional assessment) may influence Frailty (***C*_33_**).

In other words, to mitigate the AKI risk level of an elderly patient, adequate enhancement of daily activities and nutrition might offer another type of treatment; those two criteria would improve an elderly patient’s overall frailty. This finding is complementary to the result of [15]. Under certain controllable circumstances, elderly patients may improve their frailty before surgery that might cause AKI complications. Thus, the DANP model may help improve patients’ awareness of daily activities and nutrition in practice.

## 4. Discussions

Healthcare decisions are complex and often involve trade-offs among multiple conflicting objectives. Using structured and explicit approaches to medical decisions involving multiple criteria could improve decision-making using a set of techniques. MCDM methods are widely used in other sectors and there has been an increase in healthcare applications [25].

Clinical examples of MCDM applications include prioritizing patients for non-urgent surgery [26], disease diagnosis and classification [27], antibiotic-resistant diseases for R&D [28], supporting patients and physicians in selecting treatments [29], and weighing up the benefits and risks of new medicines to support licensing decisions [30]. From the clinical viewpoint, MCDM models are devised to serve as decision aids. After referring to an MCDM model’s outcome, DMs could deliberate on which might be the most beneficial option before making their final decision.

In Table 6, we transformed ten doctors’ opinions into a DANP-based AKI risk assessment model. Once an elderly patient was under evaluation, he/she could be graded from 1 (Low Risk) to 10 (High Risk) on each criterion by doctors. The overall AKI risk level of an elderly patient can be aggregated by adopting the DANP weights (in Table 5), also called the simple additive weighting (SAW) method. This model may be deployed to accumulate clinical prognoses to associate the overall AKI risk level with doctors’ evaluations. For instance, if there were three elderly patients (i.e., *A*, *B*, and *C*) who need to be assessed, their associated grades on each criterion are assumed in Table 7.

Table 7 shows that the three patients’ AKI risk levels are ranked as: A≻B≻C (i.e., *A* has the highest AKI risk), by using the DANP-based decision model. Though the model can be applied to show the ranking of patients’ AKI risk level, it is insufficient to suggest a threshold that could be regarded as dangerous. However, suppose a hospital may adopt this approach to accumulate records and doctors’ prognoses; it may collect cases for further analytics. In that case, this approach might conclude with a meaningful threshold or interval as a warning indicator. For instance, if only case *A* (in Table 7) was diagnosed as dangerous by doctors, the model may keep one record categorizing the risk level 6.50 as dangerous. This approach provides a systematic way to collect AKI-related measures for elderly patients.

Based on the group-decision theory [16], the current model can be adjusted using doctors’ experience to re-calculate the DANP weights. Take the ten doctors involved in this study as an example; they can be grouped into three categories based on their clinical experience: (1) higher than 15 years, (2) between 10 to 15 years, and (3) less than ten years.

A hospital may follow its policy to give the associated weighting to each category. Assume that the three types were assigned 50%, 30%, and 20%, respectively; the re-calculated (experience-weighted) DANP weight for each criterion will be changed accordingly. Table 8 shows the ten doctors’ numbers in each category and Table 9 compares the re-calculated DANP weights with the original ones (refer to Table 6).

The re-calculated (experience-weighted) DANP weights reveal the same ranking order for the top four criteria (i.e., $C_{33}≻C_{34}≻C_{31}≻C_{32}$); however, the remaining criteria ranking orders are not entirely the same. The experience-weighted method can be applied to follow a hospital’s policy, which is more flexible in practice.

In this illustration, we use assumed cases to illustrate how to adopt the DANP model to evaluate patients. Once an elderly patient was regarded as risky by using the model, the DEMATEL analysis findings may further guide to improving their overall AKI risk level. For instance, in Table 7, case *A*’s score on Frailty (***C*_33_**) was 9.00 (very high). Based on the findings of DEMATEL analytics (refer to Figure 2 and Table 4), Activities of daily living (***C*_31_**) and Nutritional assessment (***C*_34_**) both would influence ***C*_33_**. Thus, case *A* may consult medical professionals proactively in those two criteria to mitigate their AKI risk level. As suggested by [15], the increased AKI rate among elderly patients worsens with higher frailty. This MCDM approach proposes an overall AKI risk assessment model and provides precautionary guidance to reduce their potential AKI risk.

The MCDM approach incorporates doctors’ clinical knowledge to form a concise AKI evaluation model for elderly patients. Its short-term drawback might be lacking evidence to validate its predictive ability. However, once a hospital collects elderly patients’ scores on those criteria and organizes progress reports consistently, the model’s predictability may be further examined by statistics.

The study population is selected voluntarily; that is, the potential participants were chosen via referrals by the nephrologists, which would introduce selection bias potentially. Hawthorne effect is inevitable since the participants made a conscious decision to be in the selected hospital. Voluntary bias which could be viewed as coming from a particular sample could contain only those participants who are actually willing to participate in the survey and who participate and find the topic particularly interesting and are more likely to volunteer for that study, the same as those who are expected to be evaluated on a positive level [31].

Finally, we evaluated only one tertiary hospital, which might have characteristics that differ from those of the general population. The study’s external validity and generalization should be further considered. Future studies using a random sampling of hospitals over a broader range of regions would make the research more discursive.

## 5. Conclusions

To conclude, the present study proposes a complementary approach to enrich meta-analysis research findings [15] regarding AKI risk assessment for elderly patients. Its contributions are threefold.

(1) Identified the influential relationships among the AKI risk assessment model for elderly patients.
(2) Obtained the influential weights of the AKI evaluation criteria from ten experienced doctors.
(3) Proposed a flexible method (experience-based weighting) to follow a hospital’s policy to form a decision support system (or termed as a knowledge-based system).
(4) This study has shed light on integrating multiple research approaches (e.g., statistics and MCDM) to assist medical decision-making in practice.

## Figures and Tables

**Table 1:** Summary of the four studies that are referred to.

Ref	Country	Database Source	Research Subjects	Mean Age of Research Subjects	Duration	Key Findings
[18]	USA	Pubmed	243 AKI 74 non-AKI	57 (AKI) 56 (non-AKI)	2007–2010 (4 years)	AKI was associated with higher frailty (Clinical Frailty Scale).
[19]	Korea	Pubmed	183 (mild frail) 199 (moderate frail) 152 (severe frail)	73.8 (mild frail) 76.5 (moderate frail) 79.0 (severe frail)	2013 (1 year)	The frailest group indicated the highest AKI risk compared with the other groups.
[20]	UK	Pubmed	31 AKI 133 non-AKI	82.6 (AKI) 81.2 (non-AKI)	2 weeks	Severe frailty was associated with AKI significantly (*p* = 0.01).
[21]	USA	Pubmed	93 (non-frail) 139 (pre-frail) 136 (frail)	68.52 (non-frail) 74.71 (pre-frail) 78.83 (frail)	2013–2014 (2 years)	Frail patients were more likely to induce AKI (*p* = 0.03).

**Table 2:** The dimensions and criteria of the AKI (acute kidney injury) risk assessment decision model.

Dimensions	Criteria	Reference
	Diabetes (***C*_11_**)	[19,20]
	Hypertension (***C*_12_**)	[19,20]
Comorbidity	Depression (***C*_13_**)	(from the doctors)
(***D*_1_**)	Malignancy (***C*_14_**)	[19,20]
	Hepatitis B/C (***C*_15_**)	(from the doctors)
	Creatinine (***C*_21_**)	[19,20]
	Estimated glomerular filtration rate (***C*_22_**)	[19]
Laboratory Values	Hemoglobin (***C*_23_**)	[19]
(***D*_2_**)	Albumin (***C*_24_**)	[19]
	Na (***C*_25_**)	[19]
Comprehensive	Activities of daily living (***C*_31_**)	[19]
Geriatric	Mid-arm circumference (***C*_32_**)	[19]
Assessments	Frailty (***C*_33_**)	[15,18,19,20,21]
(***D*_3_**)	Nutritional assessment (***C*_34_**)	[19]

**Table 3:** Initial average matrix $A^{I}$.

	*C*_11_	*C*_12_	*C*_13_	*C*_14_	*C*_15_	*C*_21_	*C*_22_	*C*_23_	*C*_24_	*C*_25_	*C*_31_	*C*_32_	C_33_	C_34_
***C*_11_**	0.00	4.00	3.00	3.00	2.00	3.00	3.00	3.00	2.00	2.00	3.00	3.00	3.00	2.00
***C*_12_**	2.00	0.00	1.00	2.00	1.00	4.00	4.00	2.00	3.00	3.00	2.00	2.00	2.00	2.00
***C*_13_**	2.00	2.00	0.00	2.00	2.00	2.00	2.00	1.00	2.00	1.00	4.00	3.00	3.50	3.00
***C*_14_**	2.00	2.00	4.00	0.00	2.50	3.00	3.00	4.00	3.00	3.00	4.00	3.50	4.00	4.00
***C*_15_**	2.00	1.00	2.00	4.00	0.00	3.00	3.00	3.00	4.00	2.00	4.00	4.00	4.00	4.00
***C*_21_**	1.00	2.50	1.00	1.00	1.00	0.00	4.00	4.00	3.00	4.00	3.50	3.50	4.00	4.00
***C*_22_**	1.00	2.50	2.00	3.00	1.50	4.00	0.00	4.00	3.50	4.00	4.00	3.50	4.00	4.00
***C*_23_**	1.00	1.50	1.00	1.00	1.00	2.00	2.00	0.00	3.00	1.00	3.00	3.00	3.00	2.50
***C*_24_**	1.00	1.00	1.00	1.00	2.00	1.00	1.00	1.00	0.00	2.00	3.00	3.50	3.00	4.00
***C*_25_**	1.00	3.00	1.00	1.00	1.00	3.00	3.00	1.00	1.00	0.00	2.00	1.00	2.00	2.00
***C*_31_**	4.00	4.00	4.00	3.50	4.00	2.00	2.00	2.00	2.00	2.00	0.00	4.00	4.00	4.00
***C*_32_**	2.00	2.00	2.00	2.00	1.50	2.00	2.00	2.00	2.00	2.00	2.00	0.00	4.00	4.00
**C_33_**	2.00	2.00	4.00	3.00	2.00	2.00	2.00	2.00	2.00	2.00	4.00	4.00	0.00	4.00
**C_34_**	3.00	3.00	3.00	3.00	3.00	3.00	3.00	3.00	3.00	3.00	4.00	4.00	4.00	0.00

**Table 4:** Directional influence analytics from DEMATEL (Decision-Making Trial and Evaluation Laboratory) analysis.

Dimensions	$r^{D}$	$d^{D}$	$r^{D}+d^{D}$	$r^{D}-d^{D}$	Criteria	$r^{C}$	$d^{C}$	$r^{C}+d^{C}$	$r^{C}-d^{C}$
					***C*_11_**	3.44	2.40	5.84	1.03
					***C*_12_**	2.87	2.96	5.83	−0.09
***D*_1_**	0.74	0.59	1.33	0.16	***C*_13_**	2.92	2.87	5.80	0.05
					***C*_14_**	3.97	2.86	6.83	1.11
					***C*_15_**	3.85	2.44	6.29	1.41
					***C*_21_**	3.43	3.21	6.63	0.22
					***C*_22_**	3.85	3.21	7.06	0.64
***D*_2_**	0.63	0.67	1.30	−0.05	***C*_23_**	2.43	3.05	5.48	−0.62
					***C*_24_**	2.42	3.17	5.59	−0.76
					***C*_25_**	2.18	2.99	5.17	−0.81
					***C*_31_**	3.98	4.01	8.00	−0.03
***D*_3_**	0.77	0.88	1.66	−0.11	***C*_32_**	2.87	4.02	6.89	−1.15
					***C*_33_**	3.39	4.22	7.61	−0.83
					***C*_34_**	3.98	4.16	8.14	−0.18

**Table 5:** Normalized dimension matrix $T_{D}^{N}$.

	*D*_1_	*D*_2_	*D*_3_
***D*_1_**	0.27	0.32	0.41
***D*_2_**	0.26	0.31	0.43
***D*_3_**	0.29	0.31	0.40

**Table 6:** DANP (DEMATEL-based Analytical Network Process) influential weights for the AKI risk assessment model for elderly patients.

Dimensions	Dimensional Weights	Criteria	DANP Weights (%)
		***C*_11_**	4.92
		***C*_12_**	6.00
**Comorbidity**	27.53%	***C*_13_**	5.83
**(*D*_1_)**		***C*_14_**	5.80
		***C*_15_**	4.98
		***C*_21_**	6.45
**Laboratory**		***C*_22_**	6.46
**Values**	31.31%	***C*_23_**	6.06
**(*D*_2_)**		***C*_24_**	6.34
		***C*_25_**	5.99
**Comprehensive**		***C*_31_**	10.05
**Geriatric**	41.17%	***C*_32_**	10.04
**Assessments**		***C*_33_**	10.59 (1st)
**(*D*_3_)**		***C*_34_**	10.48 (2nd)

**Table 7:** DANP-based AKI risk assessment model with three assumed cases.

DANP			C_11_	C_12_	C_13_	C_14_	C_15_	C_21_	C_22_	C_23_	C_24_	C_25_	C_31_	C_32_	C_33_	C_34_	Risk Level
Weights (%)			4.92	6.00	5.83	5.80	4.98	6.45	6.46	6.06	6.34	5.99	10.05	10.04	10.59	10.48	
	**A**		9.00	8.00	8.00	2.00	1.00	6.00	5.00	4.00	6.00	7.00	8.00	8.00	9.00	6.00	6.50
	**B**		2.00	7.00	8.00	2.00	1.00	4.00	3.00	4.00	5.00	7.00	5.00	8.00	7.00	5.00	5.16
	**C**		7.00	6.00	2.00	2.00	1.00	5.00	2.00	3.00	2.00	4.00	7.00	5.00	5.00	5.00	4.25

**Table 8:** The number of the ten doctors in three categories.

Groups	Clinical Experience	Number of Doctors	Weighting
***G*_1_**	>15 years	1	50%
***G*_2_**	10 to 15 years	5	30%
***G*_3_**	<10 years	4	20%

**Table 9:** Comparison of the original DANP weights and the re-calculated (experience-weighted) ones.

Original	*C*_11_	*C*_12_	*C*_13_	*C*_14_	*C*_15_	*C*_21_	*C*_22_	*C*_23_	*C*_24_	*C*_25_	*C*_31_	*C*_32_	*C*_33_	*C*_34_
*DANP* (%)	4.92	6.00	5.83	5.80	4.98	6.45	6.46	6.06	6.34	5.99	10.05	10.04	10.59	10.48
(Rank)	(14th)	(9th)	(11th)	(12th)	(13th)	(6th)	(5th)	(8th)	(7th)	(10th)	(3rd)	(4th)	(1st)	(2nd)
Re-calculated *DANP* (%)	4.96	5.89	6.00	5.23	4.58	6.41	6.41	6.03	6.42	6.20	10.28	10.16	10.87	10.57
(Rank)	(13th)	(11th)	(10th)	(12th)	(14th)	(6th)	(7th)	(9th)	(5th)	(8th)	(3rd)	(4th)	(1st)	(2nd)

## Data Availability

All data underlying the findings are within the paper.

## Appendix A

**Table A1:** The dimension matrix $T_{D}^{}$.

	*D*_1_	*D*_2_	*D*_3_
***D*_1_**	0.197	0.239	0.307
***D*_2_**	0.163	0.196	0.266
***D*_3_**	0.226	0.237	0.309

**Table A2:** DEMATEL-adjusted initial supermatrix $W^{adj}$.

	*C*_11_	*C*_12_	*C*_13_	*C*_14_	*C*_15_	*C*_21_	*C*_22_	*C*_23_	*C*_24_	*C*_25_	*C*_31_	*C*_32_	*C_33_*	*C_34_*
***C*_11_**	0.036	0.053	0.051	0.048	0.049	0.046	0.043	0.047	0.046	0.044	0.055	0.054	0.051	0.054
***C*_12_**	0.065	0.049	0.059	0.056	0.053	0.064	0.059	0.059	0.054	0.070	0.062	0.063	0.059	0.062
***C*_13_**	0.059	0.054	0.045	0.065	0.057	0.053	0.055	0.054	0.053	0.051	0.062	0.062	0.069	0.061
***C*_14_**	0.059	0.061	0.058	0.045	0.066	0.053	0.059	0.054	0.053	0.051	0.059	0.062	0.063	0.061
***C*_15_**	0.047	0.047	0.052	0.051	0.040	0.046	0.046	0.047	0.054	0.045	0.055	0.051	0.051	0.054
***C*_21_**	0.068	0.070	0.068	0.065	0.065	0.048	0.068	0.067	0.064	0.076	0.063	0.063	0.063	0.063
***C*_22_**	0.068	0.070	0.068	0.065	0.065	0.069	0.049	0.067	0.064	0.076	0.063	0.063	0.063	0.063
***C*_23_**	0.065	0.056	0.059	0.067	0.064	0.067	0.066	0.048	0.061	0.056	0.060	0.060	0.060	0.060
***C*_24_**	0.062	0.064	0.068	0.064	0.070	0.063	0.065	0.075	0.054	0.058	0.062	0.062	0.062	0.062
***C*_25_**	0.059	0.062	0.058	0.061	0.058	0.066	0.065	0.056	0.069	0.047	0.059	0.059	0.059	0.059
***C*_31_**	0.103	0.101	0.106	0.102	0.101	0.103	0.105	0.105	0.101	0.107	0.080	0.095	0.105	0.104
***C*_32_**	0.103	0.101	0.099	0.099	0.102	0.103	0.102	0.106	0.106	0.096	0.105	0.078	0.105	0.105
***C_33_***	0.107	0.106	0.106	0.106	0.105	0.110	0.110	0.110	0.105	0.112	0.109	0.114	0.082	0.109
***C_34_***	0.099	0.105	0.102	0.105	0.104	0.109	0.109	0.104	0.113	0.111	0.107	0.113	0.108	0.083
